# Therapeutic antibodies for the prevention and treatment of cancer

**DOI:** 10.1186/s12929-024-00996-w

**Published:** 2024-01-12

**Authors:** Mukesh Kumar, Akansha Jalota, Sushil Kumar Sahu, Shabirul Haque

**Affiliations:** 1 Department of Medical Oncology, Thomas Jefferson University, Philadelphia, PA 19107 USA; 2 Department of Inflammation and Immunity, Lerner Research Institute, Cleveland, OH USA; 3 Department of Zoology, Siksha-Bhavana, Visva-Bharati, Santiniketan, West Bengal India; 4 Center of Autoimmune Musculoskeletal and Hematopoietic Disease, Feinstein Institute for Medical Research, Northwell Health, 350 Community Drive, Manhasset, NY 11030 USA

**Keywords:** Antibody, Cancer therapy, Antibody–drug conjugate (ADC), Immune-modulation, Chemo-resistance

## Abstract

The developments of antibodies for cancer therapeutics have made remarkable success in recent years. There are multiple factors contributing to the success of the biological molecule including origin of the antibody, isotype, affinity, avidity and mechanism of action. With better understanding of mechanism of cancer progression and immune manipulation, recombinant formats of antibodies are used to develop therapeutic modalities for manipulating the immune cells of patients by targeting specific molecules to control the disease. These molecules have been successful in minimizing the side effects instead caused by small molecules or systemic chemotherapy but because of the developing therapeutic resistance against these antibodies, combination therapy is thought to be the best bet for patient care. Here, in this review, we have discussed different aspects of antibodies in cancer therapy affecting their efficacy and mechanism of resistance with some relevant examples of the most studied molecules approved by the US FDA.

## Introduction

**Fig. 1:**
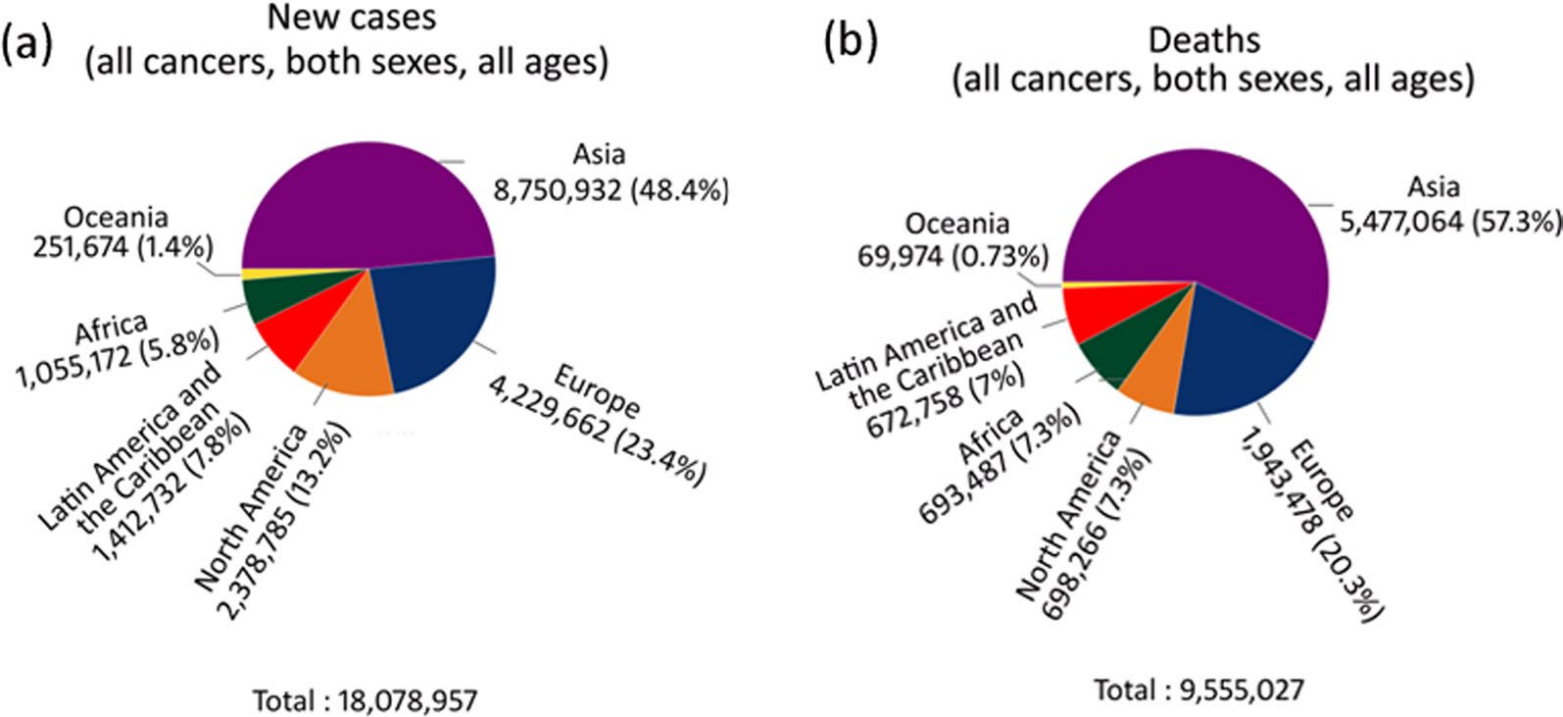
Estimated number of cancer new cases and death in 2018 by World Health Organization (WHO). For 2018, the estimated number of new cancer cases and death due to the malignancy in the world was 18 and 9.5 million respectively

Cancer is the second leading cause of death worldwide and claims approximately one out of six deaths. The heterogeneity in cancer with complex and immuno-suppressive tumor microenvironment is the real challenge to treat the disease. Acquired therapeutic resistance and the process of metastasis further aggravates the outcome due to poor prognosis and so accounts for major cause of cancer related deaths [18]. Despite our growing understanding of the disease, the worldwide diagnosed new cases and deaths are expected to increase in the future. For 2018, International agency for Research on cancer (IARC), estimated 18.0 million new cases and 9.5 million deaths worldwide (Fig. 1). Nonetheless, this number is expected to increase gradually up to 27.5 million new cases and 16.3 million deaths in year 2040.

Treatment of cancer mostly involves combination of surgery with chemo or radiation therapy. To control the side effects of conventional systemic chemotherapy, targeting molecules are prescribed to block cell proliferation and/or modulate immune response of patients having significant impact on our existing therapeutics in cancer care. Monoclonal antibodies (mAbs) are very important entities out there in the market along with small molecule inhibitors (SMIs) for targeted therapy of cancer, the former with a better specificity coupled with biological activity. The larger size of the antibodies minimizes the unwanted diffusion through plasma-membrane associated with small molecules playing a crucial role in specific targeting of the biomolecules avoiding the side effects [16, 39, 47]. The global monoclonal antibody therapeutics market was estimated at $100B USD in 2017 which is expected to reach around $219B USD by 2023 growing at a CAGR of around 12.5% during 2017 to 2023 (Zion market research).

**Fig. 2:**
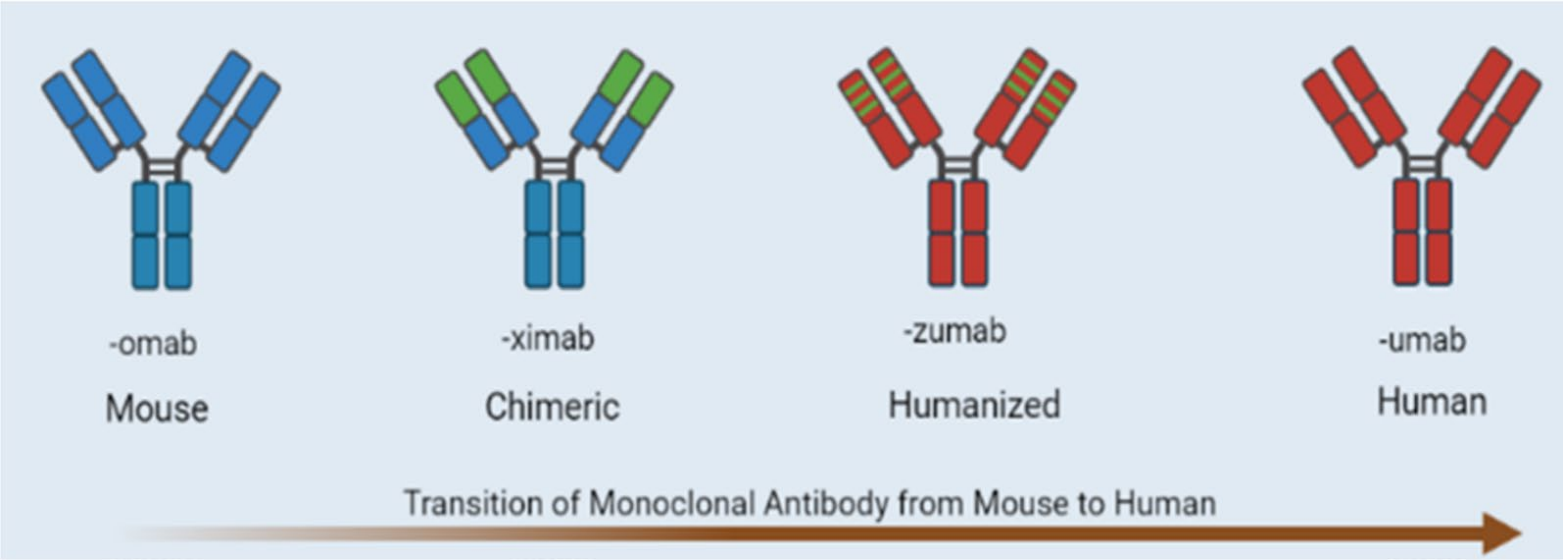
Transition of Monoclonal antibody from mouse to human. **Murine**: These antibodies originate in mice and so are mice proteins. The names of these molecules end in -omab. **Chimeric**: These are engineered antibodies in which constant regions of both chains are of human origin; however, the variable domain origin is different but not synthesized. The names of the modality end in -ximab. **Humanized**: These engineered molecules have everything of human origin except CDR regions of variable domain of both the chains that might be synthesized one too. The names of the treatments end in –zumab. **Human**: These are fully human proteins and the names of the molecules end in -umab

Paul Ehrlich’s concept of “magic bullet”, originated back in nineteenth century, inspired many others leading to the discovery of antibody’s ability to recognize the target antigen on the cell surface without harming the individual. The effort of using antibody for cancer treatment started with immunization of animals but the attempt to get anti-sera with some degree of cancer specificity could not get much success [45]. The development of inbred mice and cytotoxic assay for cell surface reactivity of alloantibodies contributed in better understanding of cell surface differentiation antigens leading to distinction between normal and malignant cells. Later, development of hybridoma technology discovered by Kohler and Milstein in 1975 met the success with analytical tools such as fluorescence-activated cell sorting (FACS) [29, 40]. The term hybridoma was suggested by Leonard Herzenberg for combining immortalization of the myeloma cells with development of selection techniques for antibody producing B cells; the two important inventions together. For the first time, antigen specific monoclonal antibodies could be developed from immortalized B lymphocytes of immunized mice spleen. The technology proved to be a weapon in dissecting the surface proteins of malignant versus healthy cells leading to greater insight into tumorigenesis [41]. But these mice monoclonal antibodies were not of much use for cancer patients because of the immunological response generating human anti-mouse antibody (HAMA) when injected in human resulting in rapid inactivation and clearance from patient serum. This also restricted multiple administration of the murine antibody required for the treatment. The advancement in recombinant technology empowered our scientists to produce chimeric, humanized, and human antibodies (Fig. 2) that geared up our fight against cancer and the current list of the FDA approved monoclonal antibodies reflects the revolution in patient care (Table 1).

**Table 1:** FDA approved antibodies for cancer treatment

Antigen	Antibody	Type	Tumor types	Year
CD20	Rituximab	Chimeric IgG1	B-cell NHL CLL	1997 2010
	Ibritumomab Tiuxetan	Mouse IgG1 Conjugated to ^90^Y	NHL	2002
	Tositumomab	Mouse IgG conjugated to ^131^I	NHL	2003
	Ofatumumab	Human IgG1	CLL	2009
	Oblinutuzumab (Glycoengineered)	Humanized IgG1	NHL,CLL	2013
CD22	Inotuzumab Ozogamicin	Humanized IgG4 conjugated to calicheamicin class of toxin (binds to minor groove of DNA)	ALL	2017
	Moxetumomab Pasudotox	Murine IgG1(dsFv)	Hairy cell leukemia	2018
CD30	Brentuximab vedotin	Chimeric IgG1 conjugated to monomethyl auristatinE (an anti-tubulin agent)	ALCL, Hodgkin lymphoma	2011
CD33	Gemtuzumab ozogamicin	Humanized IgG4 conjugated to Calicheamicin (1st FDA approved Ab-conjugate)	Acute myeloid leukemia	2000
CD38	Daratumumab	Human IgG1	Multiple myeloma	2015
	Isatuximab	Chimeric IgG1	Multiple myeloma	2020
CD19/CD3	Blinatumomab	Bispecific T-cell engager (BiTES)	ALL	2014
CD3/BCMA	Teclistamab	Bispecific T-cell engager (BiTES)	Relapsed multiple myeloma	2022
B- cell maturation antigen (BCMA)	Belantamabmafodotin	Humanized IgG1 conjugated to auristatin F	Relapsed multiple myeloma	2020
CD19	Tafasitamab	Humanized IgG1	Diffuse large B-cell lymphoma	2020
	Loncastuximabtesirine	Humanized IgG1 conjugated to pyrrolobenzodiazepine (PBD)	Diffuse large B-cell lymphoma	2021
CD52	Alemtuzumab	Humanized IgG1	CLL	2001
HER2	Trastuzumab	Humanized	Metastatic breast cancer, gastric cancer	1998
	Ado-trastuzumabemtansine	Humanized	Metatstatic breast cancer	2013
	Pertuzumab	Humanized	Metastatic breast cancer	2012
PD-L1	Atezolizumab	Humanized IgG	Urothelial carcinoma, metastatic non-small cell lung cancer	2016
	Avelumab	Full human	Metastatic markel cell carcinoma	2017
	Durvalumab	Full human	Urothelial carcinoma	2017
EGFR	Cetuximab	chimeric	Colorectal cancer	2004
	Panitumumab	Fully human (IgG2)	Colorectal cancer	2006
	Necitumumab	Fully human	Non-small cell lung carcinoma	2015
EGFR and mesenchymal- epithelial transition (MET)receptor	Amivantamab	Human bispecific IgG1	NSCLC wit EGFR exon 20 insertion mutation	2021
VEGF	Bevacizumab	Humanized	Lung cancer, glioblastoma, colorectal cancer	2004
VEGFR2	Ramucirumab	Fully human	Gastric cancer, colorectal cancer, hepatocellular carcinoma	2014
CTLA-4	Ipilimumab	Fully human	Metastatic melanoma	2011
	Tremelimumab	Human IgG2	Hepatocellular carcinoma	2022
PD-1	Pembrolizumab	Humanized IgG4	Metastatic melanoma, NSCLC, stomach cancer	2014
	Nivolumab	Human IgG4	Melanoma, lung cancer, colon cancer, liver cancer, Hodgkin lymphoma	2014
	Cemiplimab	Human IgG4	Myeloma, lung cancer, CSCC	2018
	Dostarlimab	Humanized IgG4	Endometrial cancer	2021
RANK-L	Denosumab	Human IgG2	Secondary bone cancer	2010
GD2	Dinutuximab	Chimeric IgG1	Neuroblastoma	2015
	Naxitamab	Humanized IgG1	Relapsed neuroblastoma in bone or bone marrow	2020
SLAMF7	Elotuzumab	Humanized	Multiple myeloma	2015
PDGFRA	Olaratumab	Human	Soft tissue sarcoma	2016
TROP-2	Sacituzumab govitecan	Humanized IgG1 conjugated to topoisomerase I inhibitor (SN-38)	Triple negative breast cancer, Emtastatic urothelial cancer	2021
Folate receptor- α (FR-α)	Mirvetuximabsoravtansine-gynx	Humanized IgG1 conjugated to DM4, tubulin-targeting compound	Ovarian cancer	2022

### Phage display technology in development of therapeutic antibody

**Fig. 3:**
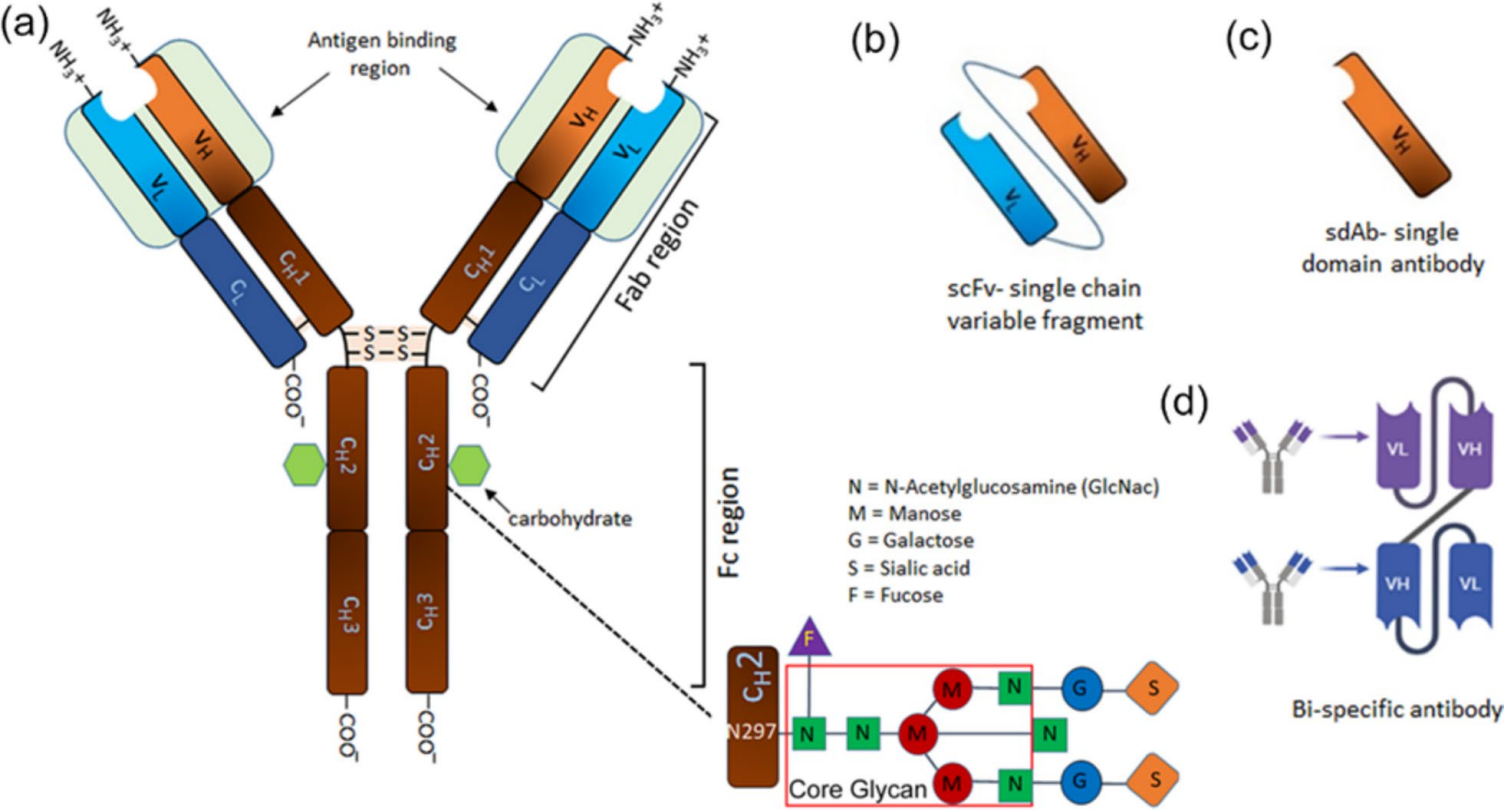
Antibody and its recombinant formats. Fab region recognizes the receptor on the cell surface and Fc region is required for effector function of the antibody. Modifications in the core glycan region is made to affect the binding with the immune cell receptors affecting ADCC, ADCP, CDC. Different small formats scFv, bispecific or singe domain antibody are used for different purposes according to the need to achieve effective therapeutic outcome

Human antibodies are the most preferred one for patient care which can be generated in transgenic animals, generally mice or rat, using hybridoma technology after introducing the human immunoglobulin loci while knocking down their own counterpart. To eliminate the necessity of injecting the antigen in animals, phage display technology, discovered in 1985 by George P. Smith, was exploited as *in-vitro* antibody selection method by Sir George P Winter which gave the privilege of easy access to the coding sequence of the antibody for further manipulation. For the discovery of this elegant method of producing pharmaceuticals, George P. Smith and Sir George P. Winter shared one-half of the 2018 Nobel Prize in Chemistry. The first human antibody Adalimumab (Humira) using phage display technology was approved in USA in 2002 for the treatment of arthritis and Crohn’s disease which inactivates tumor necrosis factor-alpha (TNF-α) [9, 28]. The human antibody display library is prepared from rearranged V-gene repertoires isolated from B-cells and/or lymphoid tissues RNA. Depending upon the need of antibody generation, the library can be naïve or immune based on the B-cells/lymphoid tissue isolation sources that can be either a healthy human or a patient. VH and VL repertoires undergoes natural *in-vivo* affinity maturation during the disease, so antibodies isolated from immune libraries have better affinity towards the disease targets while the naïve library can be used for selection against any antigens. To increase the variability of the library, which is important for selecting antibodies against diverse antigens, B-cells or tissues from multiple individuals are pooled together considering the type of library. Antibodies against toxic or non-immunogenic antigens can also be generated by this method [1, 25]. However, these antibodies are produced in *E. coli* where they are not glycosylated which might affect their binding and pharmacokinetics once expressed in eukaryotic system where post-translational modifications like glycosylation occurs. To overcome the problem, eukaryotic display systems like yeast display and mammalian cell display systems were developed [3, 49]. Recently, Rafteryet. al., has reported a workflow called PhageXpress for rapid selection of phage display library combined with Oxford Nanopore Technology, MinION sequencer. The high throughput technology uses electro-hydrodynamic manipulated solution for selection of enhanced target binders and minimizes non-specific interactions [36]. Development of recombinant technology made it easy to further manipulate the antibody formats based on requirements e.g., single-chain variable fragments (sc-Fv), diabodies (bivalent scFvs), single domain antibodies (sDAb or Nanobodies), Fabsetc (Fig. 3) which is gradually changing the spectrum of therapeutic modalities in cancer treatment and beyond.

### Factors affecting antibody therapeutic efficacy

**Fig. 4:**
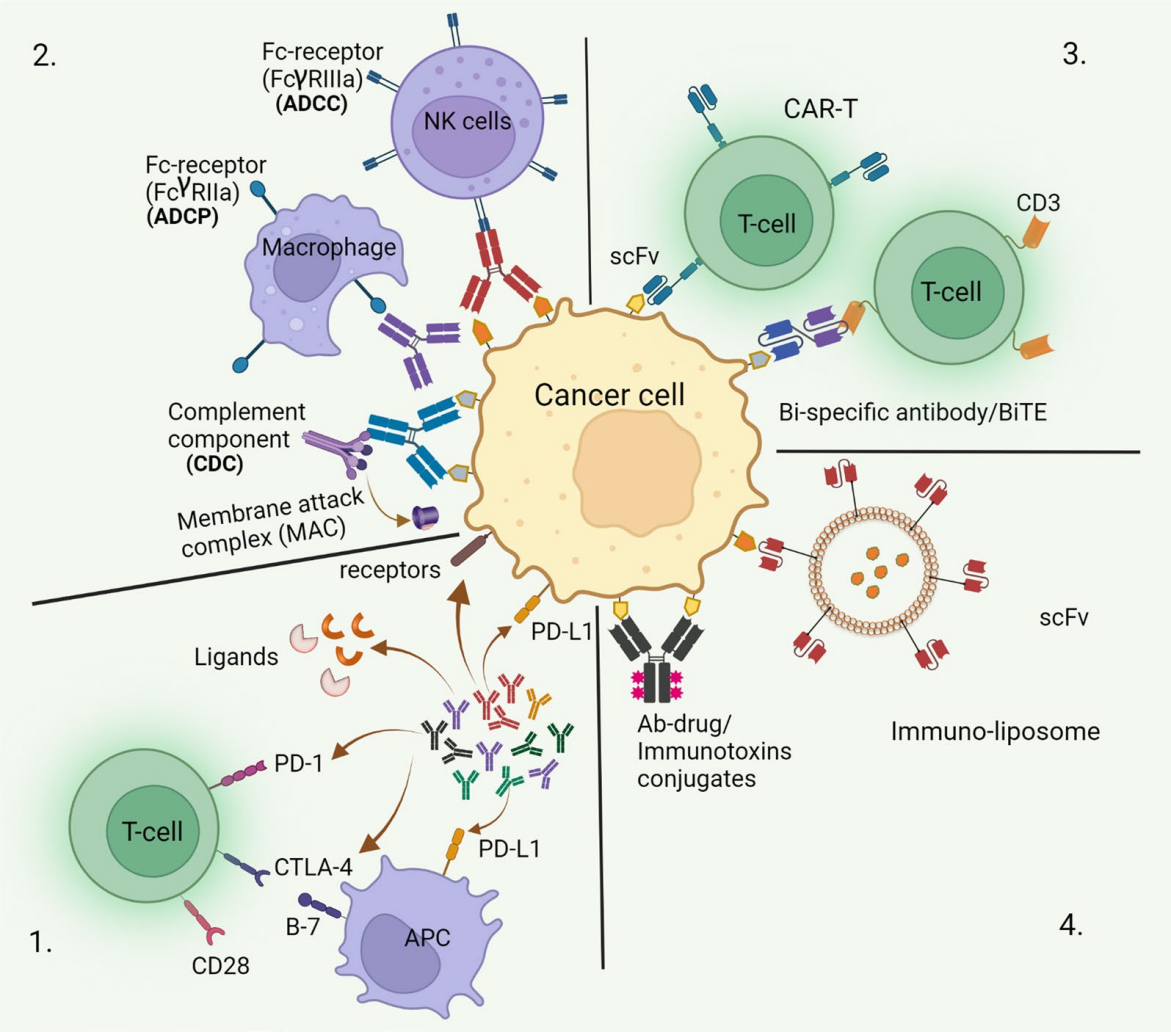
Antibody mode of action. 1. Antibodies directly bind to the receptors on the cell surface and blocks the signaling pathway required for cancerous growth, induce apoptosis or block binding of the legend inducing uncontrolled cell growth. Some of these antibodies inhibit binding of immune cell receptors to cancer cells e.g., PD-L1, PD-1, called immune checkpoint inhibitors. 2. Others, require binding of complement components or immune cells to the Fc region of the antibody to eliminate cancerous cells through CDC, ADCC and ADCP. 3. Recombinant formats (scFv or bispecific antibodies) are used for activation of T cells for cancer therapy. 4. The antibody can be loaded with cytotoxic payloads either as antibody drug-conjugates (ADC) or can be incorporated in liposomes or virosomes or exosomes surface for specific delivery of the cargo

Therapeutic antibodies targeting cancer cells bind specifically to the cell surface proteins ectopically expressed on the malignant cells (tumor-associated antigens) through variable region of Fab domain. The mere binding is not always sufficient to execute all the biological functions and so binding of the other components to Fc-region of the antibody broadens functionality of the molecule boosting therapeutic efficacy (Fig. 3). The constant regions of heavy chains, which form crystallizable fragment (Fc), play important role in effector function of the antibodies where immune cells expressing Fc-receptor (FcR) or complements bind and regulate the immune response. The direct mode of action involves binding of the antibody to the target cell-surface receptor leading to interruption of the signaling pathway or stimulating functions culminating in apoptosis induction with inhibition of cell proliferation. When antibody needs support of other component which binds to the Fc-region of the antibody, it is considered as indirect function. Depending upon the binding components, the mechanism of this indirect way of functioning can either be antibody-dependent cellular cytotoxicity (ADCC) or antibody-dependent cellular phagocytosis (ADCP) or complement dependent cytotoxicity (CDC) (Fig. 4). ADCC and ADCP varies in the FcγR-types interacting with Fc region of the antibody. NK cells are the major immune cell population participating in ADCC where FcγRIIIa binds to the cell bound antibodies and release of perforin and granzymes lyses the target cells whereas in ADCP, macrophages expressing activating FcγRIIa interacts with the Fc region of the antibody bound opsonized target culminating in phagocytosis of the target cells. For CDC, activation of compliment cascades and binding of their component to the cell bound antibody is required leading to membrane disruption and lysis of the target. These binding components help to execute the function based on the avidity of the multivalent antigen–antibody interactions readout [40, 41]. Out of the five isotypes of the antibodies based on the heavy chains (IgG, IgD, IgE, IgA, IgM), IgG is the most abundant antibody class in our blood serum which is the most common format for therapeutic antibodies. The isotypes of antibodies are defined by the Fc-region of the heavy-chain which is composed of non-covalent association of CH2 and CH3 with critical residues near hinge region. The interaction between Fc-region of the IgG antibody and the Fcγ-receptors (FcγRs) (type 1 FcR) present on immune cells (e.g., natural killer cells, macrophages, monocytes etc.) mediates cellular effector functions killing the cancer cells [2]. It is the CH2 domain of the IgG which binds to FcγR of the effector cells or complements to attack the target with different mechanism depending on the binding components. Based on the sequence of the IgG heavy-chain subclass (IgG1, IgG2, IgG3, IgG4), CH2 domain of the Fc-fragment affects the Fc-FcγR engagement and so differential effector functions. The sequence homology among these subtypes is 90% with major differences in hinge region and CH2 domain. More precisely, IgG1 antibodies contain an N-glycosylation site at highly conserved asparagine-297 (N297) residue in the CH2-domain which affects the Fc-mediated effector functions. The binding conformation of the Fc-domain is regulated by a core hepta-saccharide glycan which gets modified (glycosylation) during malignancies affecting the Fc-FcγR interaction (Fig. 3) and so the immunological response [55, 56]. The abundance of IgG subtypes depends on the cytokine milieu and nature of the antigen as well, where the latter can induce glycan modification on the specific antibody regulating the downstream response. Engineering the IgG Fc to engage the activating type 1 FcγR (FcγRI, FcγRIIa, FcγRIIIa) or genetic manipulation of inhibiting type 1 FcγR (FcγRIIb) on effector cells can modulate the cytotoxic effect of anti-tumor antibodies independent of the cell type recognized by Fab domain. Tafasitamab, an antibody against CD19, affinity for FcγRIIIa binding was improved by Fc- engineering (mutation S239D/I332E) to enhance ADCC [27]. Alteration in IgG glycosylation has significant effect on effector functions (ADCC, CDC) of the antibody. Although Fab mediated pathways e.g. induction of apoptosis by rituximab or downregulation/ blocking of receptor mediated pathways by transtuzumab/cetuximab along with Fc mediated pathways e.g. CDC or ADCC, are proposed mechanism based on the in-vitro experiments, the engagement of activating type 1 FcγR has been shown to be an absolute requirement for tumor targeting antibodies capable of depleting the target cells in-vivo including rituximab (anti-CD20), trastuzumab (anti-HER/neu), and cetuximab (EGFR) [5, 32, 35]. Absence of fucose core or presence of galactose induces ADCC, CDC, and ADCP [42, 54]. Obinutuzumab (anti-CD20) antibody, approved by FDA in 2013, was engineered for reduced fucosylation (a fucosylated antibody) to increase ADCC with enhanced binding to activating type 1 FcγIIIa receptors present on NK cells. This was achieved by expressing the antibody in an engineered eukaryotic cell line (CHO) with blocked fucosylated oligosaccharides formation and this modification extended the survival of chronic lymphocytic leukemia (CLL) patients by a year in comparison to rituximab which was unmodified anti-CD20 monoclonal antibody [6, 11]. However, a recent study has shown that Obinutuzumab, a glycoengineered antibody, can bind to only one binding site of CD20 dimer and does not facilitate complement activation whereas Rituximab and ofatumumab binds to two binding sites formed by dimerization of the antigen CD20, each Fab from two different molecules, facilitating avidity interactions and complement activation. This shows potential of Fab homotypic interactions for avidity-engineering of therapeutic antibodies rather than Fc-Fc interactions [24].

Although all these IgG variants bind to FcγRs, IgG1 is used mostly for cancer therapy because it has the highest affinity to all FcγRs for mediating ADCC. IgG1 and IgG3 both are also able to fix complement to execute CDC, but the latter has serum half-life of 5–7.5 days relative to the 21 days half-life of human IgG1, IgG2 and IgG4. Neonatal FcR (FcRn), totally unrelated to the classical FcRs but structurally related to the family of MHC class I, binds to the specific residues of the antibodies located near CH2-CH3 junction and regulates IgG half-life. Fc sialylation has also been shown to increase serum half-life of therapeutic antibodies [4]. The increased risk of immunogenicity due to significantly greater polymorphism of the long hinge region of IgG3 which is also subject to proteolysis and short serum half-life requiring frequent administration of the biological drug have restricted the therapeutic use of this subclass of IgG [25]. However, IgG2 and IgG4 have very low affinity for activating FcRIIIa in comparison to IgG1 and cannot fix complement and so cannot recruit immune cells in effector functions [15]. With increasing number of IgG4 isotype, (e.g., PD-1 antibodies) it forms the second largest subclass after IgG1 for approved therapeutic antibodies and most of these IgG4 antibodies have constant heavy chain modification. Recent studies indicate weak effector function associated with this subclass [8].

### Antibody as a drug targeting modality

Antibodies are also used to control the side effects of payloads (drugs, toxins or cytokines) administered for therapeutic purposes. Such armed antibodies are targeted to the antigen ectopically expressed on the surface of the cancer cells. These antigens may or may not have biological roles in cancer progression unlike the targets for naked antibodies. The antigen–antibody interaction induces receptor-mediated antibody internalization culminating in antibody endocytosis.

**Fig. 5:**
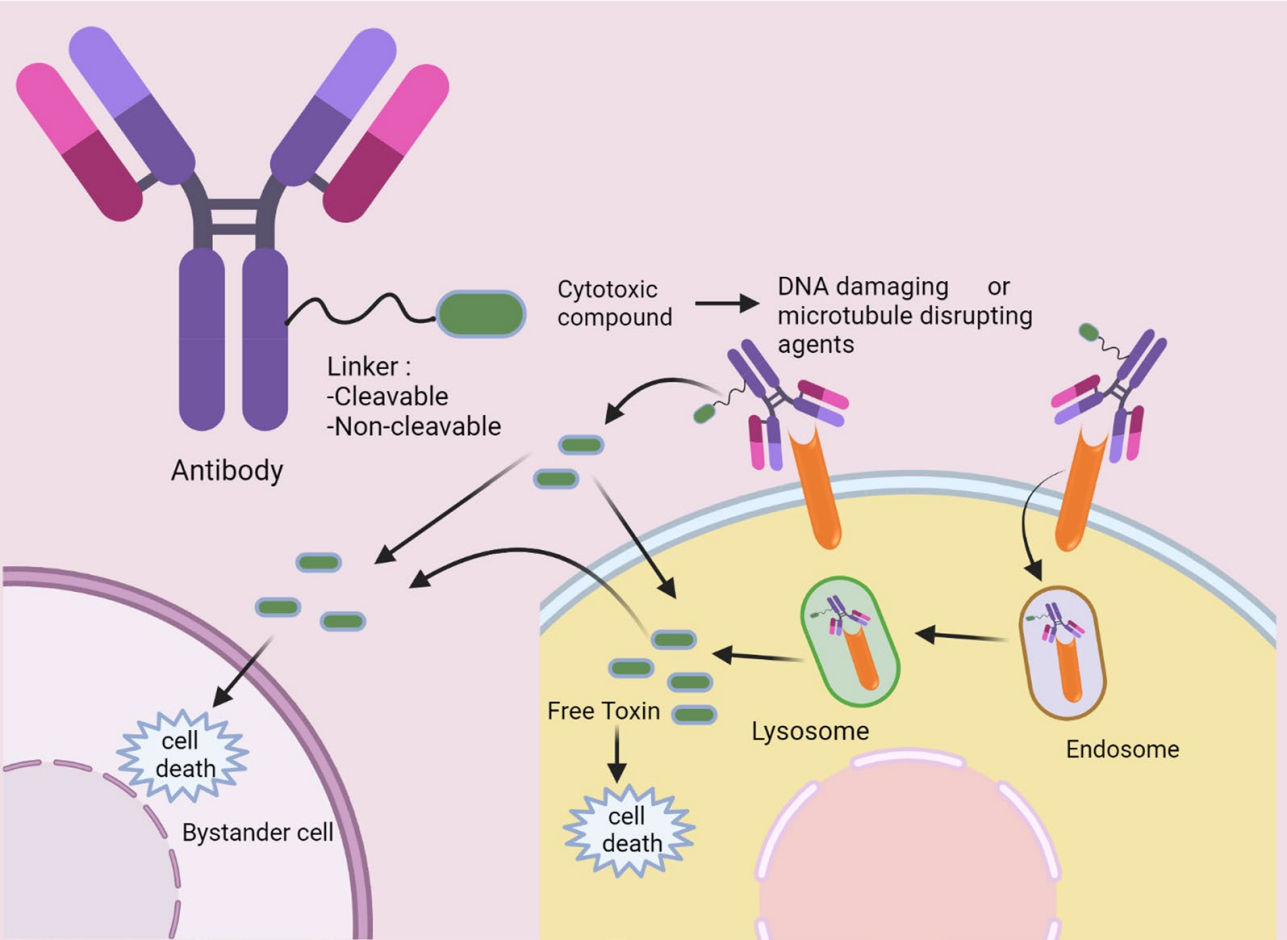
Antibody drug conjugate (ADC). The cytotoxic payload is attached to the antibody with a linker. The antibody recognizes the antigen on the cell surface and is internalized through endocytosis. In the lysosome the linker is cleaved, and the payload is released in the cytosol which is toxic to the cell. The free payload or released drug in the cytosol can also diffuse out of the target cells which can lead to bystander effect along with the drug released after cleavage of the linker once the antibody is bound to the target antigen but not internalized into the cell

For antibody targeted drug delivery, an ideal antigen should have significant differential ectopic expression on the surface of malignant cells in comparison to the non-malignant one. The effectiveness of these modalities depends on antigen characteristics and expression. The antigen should have minimum or no shedding in blood to avoid antigen–antibody binding in blood circulation which might reduce the therapeutic efficacy of the antibody on the intended site. Also, the antigen should be highly and homogeneously expressed on tumor surface with an ability to internalize the antigen–antibody complex through receptor-mediated endocytosis. Here, binding affinity of the antibody along with density of the antigen is important for these modalities as the rate of internalization of the antigen–antibody complex affects the therapeutic efficacy. Different antibody–drug conjugates (ADC) have been shown to be effective in different range of antigen density depending upon the characteristics of the antigen. For example, Gemtuzumabozogamicin (anti-CD33) has been shown effective in a range of 5000 to 10,000 receptor/cell; however, trastuzumabemtansine (T-DM1) requires more than 2 million receptors/cell. Therapeutic efficacy of such modalities is not always positively correlated with the presence of higher density of the antigen on cell surface, but it is the rate of cellular internalization of antigen–antibody complex which determines the cytotoxicity depending upon renewed expression and continuous loop of antigen internalization [7, 13, 17] (Fig. 5).

Based on the expression level of tumor associated antigen, “linker stability” and associated “bystander effects” of the released payload are two important aspects to be considered to maintain the effectiveness of the modality. Bystander killing of the neighboring cells happens with the released drug in extracellular space either before or after cellular internalization of the antibody by linker-cleavage. The membrane permeability of the endocytosed payload or its metabolite depend on hydrophilicity of the molecules adding to the bystander effect which is advantageous for targeting heterogeneously expressed tumor antigen. ADCs with cleavable-linkers, which include chemically labile (di-sulphide and pH dependent) and enzyme labile (peptide-based) linkers, will have associated bystander effect and so is used for heterogeneously expressed tumor antigens.

Studies also indicate that cellular internalization of such ADCs are dispensable for achieving the desired cytotoxicity as cleavage of the linker may occur in the more acidic tumor-microenvironment because of enhanced glycolysis and lactate generation in comparison to the normal tissue. Additionally, enhanced Cathepsin B production by tumor and associated cells as well as reducing tumor environment due to release of thiols by dead tumor cells can be reasoned for cleavage of peptide and di-sulphide bonds of drug conjugates respectively [44].

Modalities with non-cleavable (commonly a thioether) linker such as ado-trastuzumabemtansine (T-DM1) are internalized and the antibody (transtuzumab) is degraded in the lysosome, rather than linker, to release the payload inside the target cell. The potent tubulin binding maytansine derivative, DM1, do not enter surrounding cells because of the positive charge on it which prevents penetration through the cell membrane. Such modalities are good for targeting high and homogeneously expressed antigen on tumor surface with reduced or minimum bystander effect [23, 44]. Since the rate of monoclonal antibody uptake is very low in tumor cells i.e., approximately 0.003 to 0.08% of injected dose per gram in a tumor, the payload should be super-toxic to eradicate maximum tumor cells even with minimum delivery efficiency. Conventional anti-tumor drugs such as doxorubicin, etoposide is not of much consideration for the purpose because of the impaired cytotoxicity under hypoxic condition prevalent in solid tumors. Mostly used payloads in ADCs affect DNA synthesis e.g., Calicheamicin and Duocarmycin (DNA damaging agents) effective for both proliferating or non-proliferating cells and microtubule disrupting agents inhibiting mitosis cell division to block cell proliferation [50].

Along with linkage stability, homogeneity of the drug conjugation and drug to antibody ratio (DAR) are also important factors which can affect the end result. DAR represents the number of drug molecules conjugated with a single antibody and is directly correlated with cytotoxicity and pharmacokinetics of the ADC. This is affected by many other variables including the site of conjugation and design of the linker. Because of increased hydrophobicity associated with higher DAR, the ADCs cytotoxicity is compromised in *in-vivo* or clinical settings which can be improved with connecting hydrophilic groups to the drug-linker such as PEG or PHF [31].

Gemtuzumabozogamicin (anti-CD33) and Inotuzumabozogamicin (anti-CD22) both used the same non-specific conjugation chemistry, but the former was withdrawn in 2010 due to associated toxicity with serious liver condition at higher dose and reintroduced in 2017 with lower recommended dose for better clinical outcome. Calicheamin, which induces double-strand DNA breaks and is a potent cytotoxic anti-tumor agent, was introduced in the clinic as Gemtuzumabozogamicin conjugate in 2000. Everything between the two conjugates (Gemtuzumab and Inotuzumab) including isotypes of IgG (IgG4) were similar except the targeting antigens. It has also been demonstrated that Gemtuzumabozogamicin induced cell death was not only Calicheamin mediated but CD33 signaling also contributed unlike Inotuzumabozogamicin where CD22 signaling had no role. However, the half-life of these conjugates was found to be different in human and is attributed to the heterogeneity of the preparation and nonspecific release of the drug in circulation [10, 46]. As host effector function is not required in ADCs mediated cytotoxicity so IgG4 isotypes can be a better choice for such entities. However, it has been shown that IgG4 has relatively low affinity for FcγR than IgG1, but it can bind with FcγRIIIa in non-fucosylated form [12]. Binding to FcγRs is not always good for ADCs but can be undesirable as in case of T-DM1 (IgG1) binding with FcYRIIa leading to thrombocytopenia [26]. So multiple factors play diverse role affecting the cytotoxicity of a modality especially in *in-vivo* or clinical settings and the strategy varies on case-by-case basis.

### Resistance for antibody therapy

Development of therapeutic antibodies has revolutionized cancer treatment, but the improved outcomes have sometimes their own challenges. Resistance against therapy is a major concern which can either be attributed to some mutation present in the tumor before start of the treatment or may be acquired under continuous immune selection pressure of the treatment resulting in subsequent alteration in signaling of the survival pathways. FDA approved Cetuximab and panitumumab both antibodies against ectodomain of EGFR targeting the same epitope for the treatment of metastatic colorectal cancer. Therapeutic resistance has been observed for cetuximab but not for panitumumab in patients with mutation (S492R) in ectodomain of EGFR which affects binding of cetuximab but not of panitumumab [30, 43]. Activating mutation in oncogenes like K-Ras, BRAF, PIK3CA have been predictive to cetuximab (anti-EGFR) resistance in colorectal cancer treatment. Mutations in HER2 does not affect the binding of transtuzumab but the tumor loses sensitivity to it because of continued activation of PI3K/AKT pathway [22, 54]. Also, any activating mutation in PI3K/AKT/mTOR pathway molecules decreases the sensitivity and increases the resistance to transtuzumab. It has been shown that such tumors can be better treated with transtuzumab in combination with PI3K/AKT inhibitor which is also effective against cancer stem cells (CSC), a population hypothesized to be responsible for therapy resistance and cancer reoccurrence [38].

Therapy resistance can also originate in tumors transitioning phenotypically from epithelial to mesenchymal (EMT) state resulting in loss of cell-to-cell contact and become more migratory in nature promoting stem cells characteristics. Resistance to cetuximab (anti-EGFR) in head and neck cancer and oral squamous cell carcinoma has been observed because of EMT transition and loss of EGFR expression was also noticed. Activation of EMT pathway has been shown to be the key predictor of cetuximab resistance in K-Ras wild-type colorectal cancer [20, 33].

Since ADCC is important for antibody mediated cytotoxicity to cancer cells where immune cells interacts with Fc domain of antibody, so alteration in expression of the interacting molecules on the immune cells surface affect the end results. Relative ratio of activating (FcYRI, FcYRIIA and FcYRIII) versus inhibiting (FcYRIIB) receptors expression on immune cells and preferred Fc domain interacting moieties determine the level of cytotoxicity. Polymorphism in FcYRIIa and/or FcYRIII affects the clinical success of rituximab, transtuzumab and cetuximab based on their affinity to Fc domain of IgG. NK cells are crucial for ADCC, and it has been shown that cross-linking of FcYRIIIA (CD16) compromises the efficacy of therapeutic antibodies in cancer [52]. Avidity i.e., cumulative effect of multiple non-covalent interactions (affinities) of antigen–antibody on the cell surface, plays an important role in deciding the threshold of antibody-based effector function activation. Amivantamab, a bispecific antibody targeting EGFR and mesenchymal-epithelial transition (MET) receptor, has a low-level core fucosylation and enhanced FcγRIIIa binding leading to ADCC induced cancer cell death. In tumors with EGFR mutation and MET mutation/amplification, a crosstalk has been reported between the two pathways and such tumors are also resistant to other available therapies. Simultaneous blocking of the two pathways by the bispecific antibody shows increased selectivity and synergistic effect which can be explained by avidity effect [53]. Lower or heterogeneous expression of antigen and inhibition of complement dependent cytotoxicity have also been reasoned for antibody mediated therapeutic resistance in cancer [37]. The former also affects antibody drug conjugates (ADC) modalities efficacy. Loss of CD30 and lower expression of HER2 has been reported for compromised efficacy of brentuximabvedotin and transtuzumab-DM1 (T-DM1) [7].

### Antibodies in immuno-modulation

Tumor microenvironment composed of extracellular matrix proteins, fibroblasts and mesenchymal stromal cells along with infiltrating immune cells, plays an important role in tumor progression by manipulating immune cells. Under the influence of tumor derived factors, these immune cells once suppressed become pro-tumorigenic rather than anti-tumorigenic. Because of the absence of activating FcγRs on T lymphocytes, antibodies cannot directly recruit T cells. However, bi-specific T cell engager (BiTE) binds to a tumor antigen at one end and simultaneously binds CD3 molecule on T cells leading to T-cell activation without co-stimulatory signal like CD28. Here, tumor cells play indispensable role which is engaged by one of the single-chain variable fragment (scFv) connected by a linker to another scFv engaging T-cell. IgG2 and IgG4 are considered for development of these modalities as IgG1 has been found toxic for activated T cells leading to elimination of the later [48]. Blinatumomab is such a BiTE approved for the treatment of acute lymphoblastic leukemia (ALL) in 2014.

Another strategy of activating T-cells against tumor comprises of genetic modification of T-cells with chimeric antigen receptor (CAR) using scFv as an antigen recognizing molecule while making the T cells cytotoxicity independent of MHC restrictions like BiTE. Kymriah (Novartis) and Yescarta (Kyte-Gilead) are two FDA approved (2017) therapies for treatment of B-cell malignancy. The strategies, being MHC independent, circumvents the escape mechanisms of tumor like downregulation of MHC I molecules, loss of co-stimulating molecules, upregulation of anti-apoptotic molecules etc. In 2020, CAR T cells (Tecartus) got FDA approval as the first cell-based gene therapy modality for the treatment of acute lymphoblastic leukemia (ALL)and mantle cell lymphoma (MCL). Apart from this, checkpoint inhibitor antibodies are used for activating endogenous T-cells against tumor e.g., CTLA-4, PD-1 antibodies etc. Studies have shown that polymorphism in FcYRIIa and FcYRIIIa affects the associated affinity for Fc and in turn, the efficacy of antibodies irrespective of tumor types and antigens illustrating the importance of ADCC in clinical success of these molecules [21].

Since avidity plays crucial role in execution of some effector functions so bi-specific and multi-specific antibody formats are having therapeutic advantages. Increasing level of target occupancy of antibody on the cell surface and so density of the antibody favoring different level of avidity triggers Fc-mediated effector function at different occupancy saturation levels [34].

### Future prospective

Recombinant constructs of antibodies targeting multiple antigens at a time (multi-specific antibody) are being developed for better efficacy. Apart from antibody–drug conjugates, antibody- small interfering RNA (siRNA) conjugates and antibody-cytokines fusion protein modalities are getting increased attention [19]. Because of the associated cytokines release syndrome, the innovative CAR T cell therapy for cancer has its own challenges. To circumvent the hurdles, researchers are trying to use extracellular vesicles mostly exosomes carrying CAR after stimulating the engineered cells (T cells, dendritic cells, NK cells etc.) with antigen. These exosomes do not express PD1 molecule and so avoids immunosuppression by cancer cells but can inhibit tumor growth in antigen dependent manner [14, 51].

## Conclusions

Antibodies are great success as a biological therapeutic drug especially in last few years for cancer therapy. The potential of these molecules is reflected in the market value of the monoclonal antibodies in cancer therapy which is expected to hit around USD 159.96 billion by 2030 from USD 62.2 billion in 2021. The major fraction of the market share is dominated by humanized antibodies (39.66% in 2021) as developing mouse monoclonal is relatively easy and cost effective. Additionally, other platforms to develop human antibodies along with genetic engineering techniques are the major driving force for the market. There is therapeutic resistance developed against some of these molecules but because of the minimum side effects, promising efficacy in combination therapy and ability to redirect the immune cells against cancer cells, more monoclonal antibodies are expected to get approval for better patient care.

## Data Availability

Not applicable.
